# The predominant expression of cancer stem cell marker ALDH1A3 in tumor infiltrative area is associated with shorter overall survival of human glioblastoma

**DOI:** 10.1186/s12885-020-07153-0

**Published:** 2020-07-17

**Authors:** Chao Gan, Daniela Pierscianek, Nicolai El Hindy, Yahya Ahmadipour, Kathy Keyvani, Ulrich Sure, Yuan Zhu

**Affiliations:** 1 Department of Neurosurgery and Spine Surgery, University hospital Essen, University of Duisburg-Essen, Hufelandstrasse 55, 45122 Essen, Germany; 2 Department of Neurosurgery, Tongji Hospital, Tongji Medical College, Huazhong University of Science and Technology, Wuhan, China; 3 Present Address: Department of Spine- and Peripheral Nerve-Surgery, St. Christophorus 625 Hospital, Werne, Germany; 4 Institute of Neuropathology, University hospital Essen, University of Duisburg-Essen, Essen, Germany

**Keywords:** Primary glioblastoma, ALDH1A3, Cancer stem cell marker, Overall survival, Peritumoral edema

## Abstract

**Background:**

ALDH1A3 is a cancer stem cell marker in neoplasms including glioblastoma (GBM). However, the comprehensive role of ALDH1A3 in GBM remains unclear. This study attempted to investigate the expression of ALDH1A3 in human GBM tissues and its association with clinical parameters.

**Methods:**

Thirty primary GBM and 9 control were enrolled in this study. ALDH1A3 mRNA and protein expression levels were detected by RT^2^-PCR and western blot, respectively. Immunohistochemistry and immunofluorescence staining were performed to evaluate the regional and cellular expression manner of ALDH1A3. The association of ALDH1A3 expression with multiple clinical parameters was analyzed.

**Results:**

ALDH1A3 protein level, but not mRNA level, in a subgroup of GBM was significantly higher than that in the control group. ALDH1A3 immunoreactivity was detected heterogeneously in individual GBMs. Fifteen of 30 cases showed a positive of ALDH1A3 immunoreactivity which was predominantly observed in the tumor infiltrative area (TI). Double immunofluorescence staining revealed a co-localization of ALDH1A3 with GFAP in glial-shaped cells and in tumor cells. ALDH1A3 immunoreactivity was often merged with CD44, but not with CD68. Moreover, ALDH1A3 expression was positively associated with the tumor edema grade and inversely with overall survival (OS) (median OS: 16 months vs 10 months), but with neither MGMT promoter methylation status nor Ki67 index in GBM. An upregulation of ALDH1A3 was accompanied by a reduced expression of STAT3β and p-STAT3β.

**Conclusions:**

Inter- and intra-tumoral heterogeneous expression of ALDH1A3 was exhibited in GBMs. A high immunoreactivity of ALDH1A3 in tumor infiltrative area was associated with shorter OS, especially in patients with MGMT promoter methylation. Our findings propose ALDH1A3 not only as a predictive biomarker but also as a potential target for personalized therapy of GBM.

## Background

Glioblastoma (GBM), the most common primary malignant brain tumor in adults, is genetically and histopathologically highly heterogeneous. A median survival period is 15 months despite the advanced treatment including surgical resection and chemoradiotherapy [1]. Increasing evidence suggests that cancer stem cells (CSCs) are crucial for tumorigenesis, therapeutic resistance and recurrence in GBM [2, 3]. Given that subventricular zone (SVZ) consists of enriched neural stem cells that possess the capacity to generate neurons and glia throughout adulthood [4, 5]. Gliomas are thus often presumed being initiated by neural stem cells in SVZ [6–8]. Indeed, expression of multiple CSC markers in GBM is negatively associated with overall survival in GBM patients [9, 10]. Therefore, targeting CSCs is considered as a promising therapeutic strategy.

Aldehyde dehydrogenases (ALDHs) are a group of enzymes consisting of 19 isoforms. Besides the metabolic functions [11, 12], high ALDH activity is considered as a hallmark of CSCs in various cancers [13]. Targeting ALDH inhibits the proliferation of GBM tumor cells and CSCs [14]. ALDH1A3 is the most upregulated between ALDH high and low subgroups of glioma cells among 19 isoform of ALDH family [15]. ALDH1A3 prominently emerges as a CSC marker to be targeted in multiple neoplasms [16–18]. Of note, ALDH1A3 is enriched in mesenchymal subtype (MES) of GBM patients, thereby being a sensitive and specific marker for MES GBM subtype [15]. ALDH1A3 is crucial for transition from proneural-CSCs to MES-CSC and is important for the maintenance of MES subtype [19]. Besides, ALDH1A3 plays also important roles in regulating self-renewal, differentiation and chemo/radio-resistance [20].

Most of the studies of ALDH1A3 expression in dataset were performed at transcriptional level by microarray in GBM. However, the comprehensive association of ALDH1A3 protein expression with clinical outcome remains elusive. The present study focused on the investigation of ALDH1A3 protein expression in a cohort of GBM patients with emphasis on its regional expression pattern and cellular localization and on its correlation with clinical parameters. We also explored the STAT3 and Akt/PTEN signaling cascades which might be involved in the regulation of ALDH1A3 expression and in its functions in GBM as studied by other entities. Through this study, we anticipate providing a broader perspective on this molecule as a prognostic biomarker as well as a potential therapeutic target for GBM.

## Methods

### Patient cohort, magnetic resonance imaging (MRI)-based edema grading and evaluation of the Karnofsky performance index (KPI)

Surgical specimens (*n* = 30) were collected from adult patients with primary GBM who were treated in the Department of Neurosurgery at our hospital. All enrolled patients were histopathologically diagnosed with primary GBM (WHO grade IV). Surgical specimens from patients who suffered temporal lobe epilepsy and underwent anterior temporal lobe resections without histopathological findings were used as control (*n* = 9).

Tumor edema appears as a region with increased T2 signal intensity outside the gadolinium-enhanced portion. Peritumoral edema is classified into four grades based on preoperative MRI scans [21]. Briefly, grade zero reflects no edema on preoperative scans; if edema is seen as less than, or approximately equal to, or greater than the tumor itself, the edema is graded as I, II, or III, respectively.

The preoperative KPI as one of the major prognostic indicators for GBM survival was used to assess all patients enrolled in the present study.

### Evaluation of O6-methylguanine-methyltransferase (MGMT) promoter methylation and IDH1 mutation

For *MGMT* promoter methylation analysis, genomic DNA was isolated from paraffin sections of GBM. *MGMT* promoter methylation was analyzed by methylation-specific PCR as described previously [22].

IDH1-R132H, the most common glioma derived mutation, was determined immuno-histochemically in paraffin-embedded tumor specimens with a specific antibody as described previously [23].

### TCGA database analysis of ALDH1A3 gene expression and OS dataset in GBM

The data of *ALDH1A3* gene expression and its association with OS in 525 GBM cases and 10 normal control from The Cancer Genome Atlas (TCGA-GBM) were obtained from the GlioVis browser [24]. The gene expression profile was measured using the Affymetrix HT Human Genome U133a microarray platform. Kaplan–Meier survival curve was generated to show patient survival status between *ALDH1A3* high and low group with optimal cutoff provided by GlioVis itself.

### Real time-reverse transcription-polymerase chain reaction (RT^2^-PCR)

The extraction of total RNA (Qiagen, Hilden) and cDNA synthesis (Bio-Rad, Munich) were performed according to the manufacturer’s instructions. The primer sequences and annealing temperatures are listed in Table 1. The following condition was used for real-time PCR: initial denaturation at 95 °C for 2 min, 35–45 cycles of amplification at 95 °C for 5 s and at annealing temperature for 25 s. Melting curve was obtained using the following settings: 95 °C for 1 min, and 55 °C for 1 min, and 55–95 °C with a heating increase rate of 0.5 °C every 10 s. Relative mRNA expression (fold change) was calculated according to the cycle threshold approach (2^-ΔΔCT^ method), and normalized to the reference gene GAPDH as described [25].

**Table 1:** Primer sequences and annealing temperatures for real-time reverse-transcription PCR (RT^2^-PCR)

Primer name	Sequence	Annealing temperature (°C)
*Nestin*		62
for.	CTCCAAGAATGGAGGCTGTAGGAA	
rev.	CCTATGAGATGGAGCAGGCAAGA	
*CD133*		60
for.	CAGAAGGCATATGAATCC	
rev.	CACCACATTTGTTACAGC	
*CD44*		60
for.	CCCAGATGGAGAAAGCTCTG	
rev.	ACTTGGCTTTCTGTCCTCCA	
*YKL40*		63
for.	GACCACAGGCCATCACAGTCC	
rev.	TGTACCCCACAGCATAGTCAGTGTT	
*ALDH1A3*		60
for.	TCTCGACAAAGCCCTGAAGT	
rev.	TATTCGGCCAAAGCGTATTC	
*OLIG2*		60
for.	CTCCTCAAATCGCATCCAGA	
rev.	AGAAAAAGGTCATCGGGCTC	
*SOX2*		64
for.	ACCGGCGGCAACCAGAAGAACAG	
rev.	GCGCCGCGGCCGGTATTTAT	

*for*. forward, *rev.* reverse

### Immunohistochemistry staining and analysis

Immunohistochemistry was performed on formalin-fixed and paraffin-embedded (FFPE) GBM sections (*n* = 30). Briefly, after deparaffinization in gradient ethanol, heat-induced epitope retrieval and blocking the unspecific binding, sections were incubated with rabbit anti-ALDH1A3 primary antibody (1:250, Novus Biologicals) overnight at 4 °C. After the incubation with a HRP-conjugated secondary antibody (1:1000, Cell Signaling Technology), the sections were incubated with the substrate 3,3’-diaminobenzidine kit (Invitrogen) followed by hematoxylin counter staining. Negative control staining was done omitting primary antibody instead of a non-specific rabbit immunoglobulin fraction (DAKO).

The ALDH1A3 immunoreactivity in all stained sections was quantified according to previous description [26]. Briefly, the intensity of ALDH1A3 immunoreactivity was scored as 0–3: 0 = negative; 1 = weak; 2 = moderate; 3 = strong. The percentage of positive cells in microscopic images (magnification × 200) was counted using ImageJ, and four categories (0–3) were defined: category 0, < 1%; category 1, 1–5%; category 2, 5–10%; category 3, > 10%. The immunoreactive score (IRS) was calculated using the following formula: the score of the immuno-intensity × the score of positive percentage. According to IRS, the patients were classified to three groups: negative/low: IRS ≤ 2; medium: IRS > 2; and high: IRS ≥ 6.

### Double immunofluorescence staining and imaging

For FFPE tissue sections, deparaffinization, heat-induced epitope retrieval, and blocking steps were performed as described previously [22]. Thereafter, the sections were incubated overnight at 4 °C in the primary antibody mixtures containing rabbit anti-ALDH1A3 (1:250) and a cell type specific marker antibody, i.e.*,* mouse anti-GFAP (1:250, Sigma Aldrich) or mouse anti-CD68 (1:100, gift from Neuropathology in our hospital) or rat anti-CD44 (1:100, Invitrogen). The sections were incubated with the mixture of biotinylated goat anti-rabbit IgG and Texas red conjugated horse anti-mouse IgG at room temperature for 1 h followed by the substrate reaction with FITC-conjugated avidin. Counter staining was done with Hoechst-33,342. Images were acquired using Axio Imager M2 microscope (Zeiss) with the ApoTome.2 system for optical sections.

### Western blot

Total protein extraction and electrophoresis were performed as before [21]. The primary antibody reaction was done overnight at 4 °C with the following primary antibodies (excepting ALDH1A3 antibody, all from Cell Signaling Technology): anti-ALDH1A3 (1:1000, Novus Biologicals), anti-STAT3 (1:1000), anti-phospho-STAT3 (Tyr705) (p-STAT3) (1:2000), anti-GAPDH (1:1000), anti-phospho-Akt (Ser437) (p-Akt) (1:1000), anti-PTEN (1:1000). After the second antibody reaction, the blots were incubated with ECL solution (GE Healthcare) and the image was acquired by using ImageQuant LAS 500 (GE Healthcare, Freiburg).

### Statistical analysis

The experimental data were presented as the mean ± standard deviation (SD). All statistical analysis were performed using the GraphPad Prism 5 and SPSS 23.0. Student t test with Welch’s correction was used for data analysis between two groups; one way ANOVA followed by Bonferroni’s multiple comparison test was applied for multi-group comparison. The survival curve was plotted using the Kaplan-Meier method and analyzed using log-rank test. A *P* value less than 0.05 was considered statistically significant.

## Results

### Baseline characteristics of patient

The mean age of GBM patients at the first diagnosis was 60.1 ± 13.3 years. The ratio of male to female was 1: 1.1 (14 to16). Among 30 GBMs, 60% (18/30) patient had a gross total resection of tumor. Twenty-six patients (86.7%) showed KPI ≥ 70. Fourteen of 30 cases (46.7%) had a positive methylation status of MGMT promoter. A R132H point mutation of IDH1 was detected in 3 of 20 cases, whereas IDH mutation information was not assessed in the other 10 cases. A standard chemoradiotherapy [1] after surgical resection was applied to 63.3% (19/30) of patients. The mean of OS was 13.3 ± 9.9 months. Given that SVZ is suggested as one of the prognostic factors in GBM [6], GBM patients were grouped upon their tumor location, whether the tumor contacted SVZ (+) or not (SVZ−). In 20 of 30 (66.7%) cases GBM tumors were found at the SVZ+ location.

### *ALDH1A3* mRNA expression was downregulated in GBM: TCGA database and own data

**Fig. 1:**
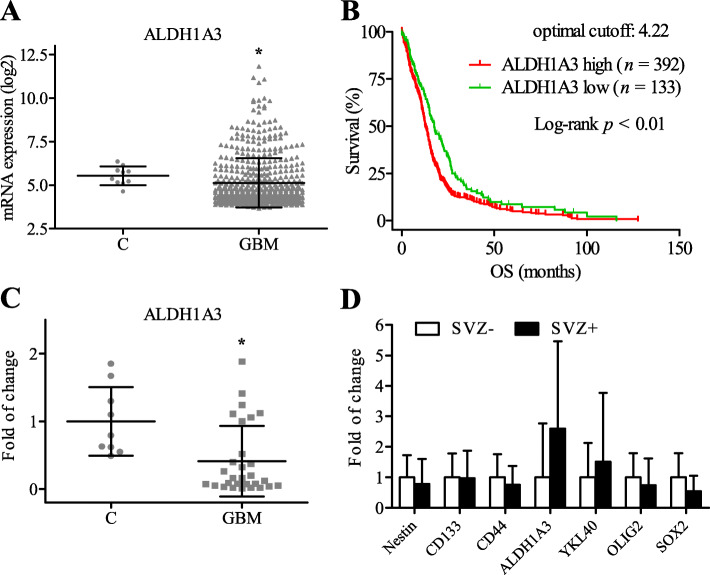
mRNA expression of *ALDH1A3* in GBM. **a** TCGA database showed a lower expression of ALDH1A3 mRNA in GBM (*n* = 525) compared with the control (*n* = 10). **b** The higher expression of *ALDH1A3* mRNA was associated with the shorter overall survival (OS) of GBM patients at optimal cutoff based on TCGA database. **c** mRNA expression of *ALDH1A3* detected in the present study by real-time RT-PCR in primary GBM (*n* = 30) and control (*n* = 9) supported the data from TCGA database. **d** Subgroup analysis of mRNA expression of *ALDH1A3* and other stem cell markers in subventricular zone (SVZ+) and in non-subventricular zone (SVZ−). Student’s t test with Welch’s correction was utilized between two groups in **a**, **c**, **d** (*P < 0.05)

Microarray-based TCGA database revealed a statistically reduced *ALDH1A3* mRNA in GBM compared to control group (*P* < 0.05, Fig. 1a) and a negative correlation between the *ALDH1A3* mRNA and the OS of GBM patients, i.e.*,* higher *ALDH1A3* mRNA expression and shorter OS with optimal cutoff of *ALDH1A3* mRNA value at 4.22 (*P* < 0.01, Fig. 1b).

To validate the finding from the TCGA datasets, RT-PCR was performed and found a significant downregulation of *ALDH1A3* mRNA expression in our patient cohort (*P* < 0.05, Fig. 1c). To compare SVZ+ GBM with SVZ− GBM in the matter of CSCs characteristics, not only *ALDH1A3* but also some other known CSC markers including *Nestin, CD133*, *CD44*, *YKL40*, *OLIG2* and *SOX2* were examined. Interestingly, the *ALDH1A3* mRNA level was 2.59-fold higher in SVZ+ group compared to SVZ−. However, neither *ALDH1A3* nor other detected CSC markers showed significant difference between SVZ+ and SVZ− groups (Fig. 1d).

### Characterization of regional and cell type specific expression of ALDH1A3 in GBM

**Fig. 2:**
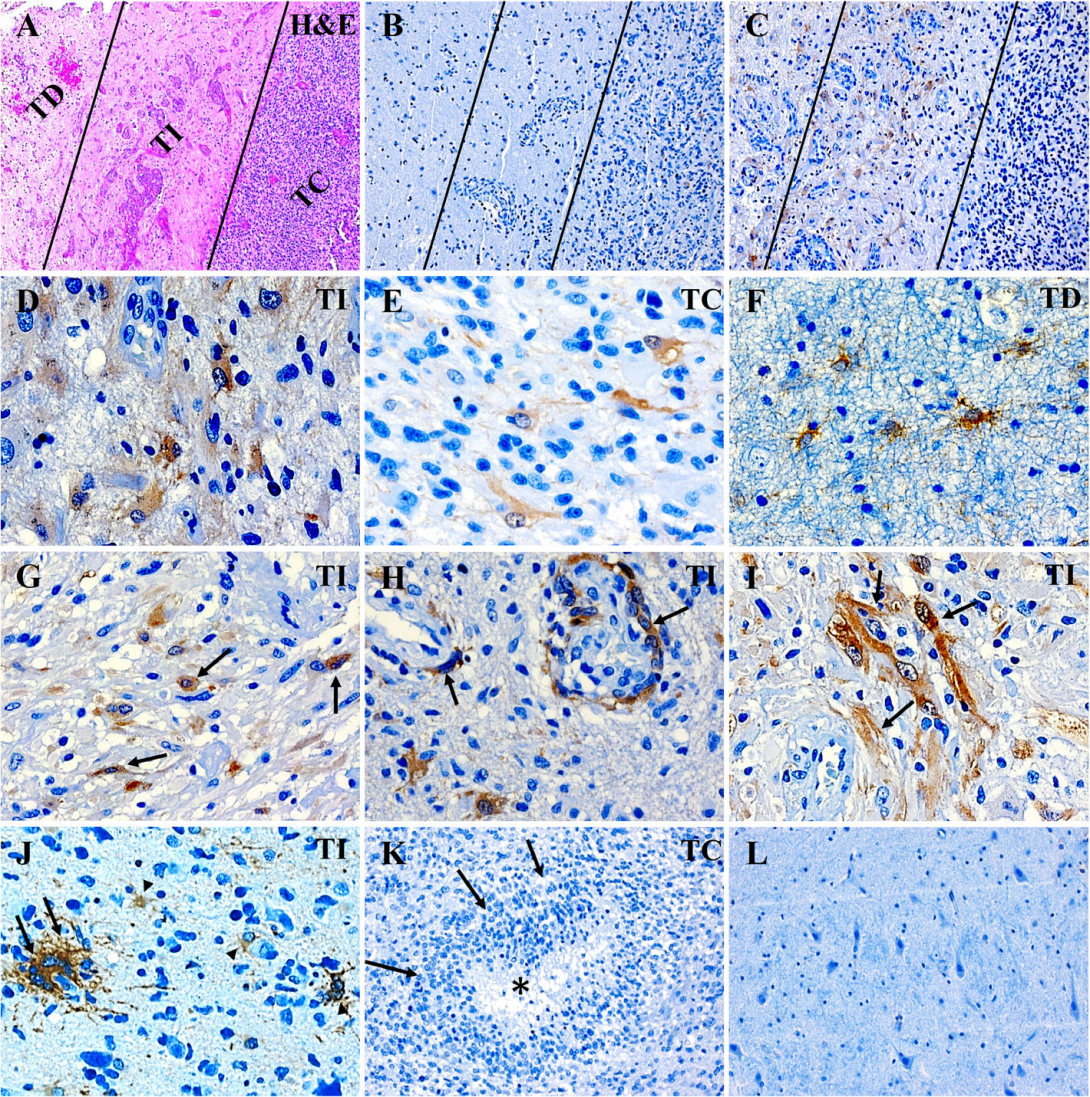
Immunohistochemistry of ALDH1A3 in GBM. **a** H&E staining defined the different regions in GBM sections. TC: tumor center; TI: tumor infiltrative area; and TD: tumor distant area. **b**, **c** Immunostaining of ALDH1A3 in GBM sections revealed heterogeneous expression of ALDH1A3 in different GBM patient sections (*n* = 30). The representative photos show the absence of ALDH1A3 expression **b** and the positive expression of ALDH1A3 **c**, respectively, in two GBM cases. The positive ALDH1A3 expressing cells were mainly detected in TI, and much less in TC. **d-f** Images are representative of the expression of ALDH1A3 in the different regions of GBM tissue. **g-k** Expression of ALDH1A3 in typical tumor cells (arrows in **g**), in out-layer of glomeruloid tufts (arrows in **h**), in some vessels (arrows in **i**), in multi-nuclear cells (arrow in **j**) and in glial-shaped cells (arrowheads in **j**). ALDH1A3 immunoreactivity was not detected in the necrosis core (asterisk in **k**) and the pseudopalisade structure (arrows in **k**). **l** ALDH1A3 immunoreactivity was not detected in the non-tumoral control brain section. **a-c**, original magnification × 100; **d-j**, original magnification × 400; **k-l**, original magnification × 200

Immunohistochemistry (IHC) revealed a heterogeneous expression of ALDH1A3 in different GBM sections and in the different regions of individual cases. To characterize its expression pattern upon area of focus, we classified tumor regions into three: tumor center (TC), tumor infiltrative area (TI) and tumor distant area (TD) (Fig. 2a, lines). TI showed a gradually lower tumor cell density in comparison to TC, whereas TD exhibited normal cells as well as some scattered tumor cells. An apparent expression of ALDH1A3 was found in 50% of GBM specimens (15/30). Figure 2b and c were representative of a negative and a positive ALDH1A3 expression case, respectively. In the cases showing ALDH1A3 expression, ALDH1A3 immunoreactivity was dominantly detected in TI (Fig. 2c, d, g-j), whereas scattered ALDH1A3 positive cells were also found in both TC (Fig. 2e) and TD (Fig. 2f). For quantitative analysis of ALDH1A3 expression, IRS was determined upon area of focus in tumor and classified patient cases into three subgroups: negative/low (IRS ≤ 2), medium (IRS > 2) and high (IRS ≥ 6). According to IRS of 30 patient cases, 24, 3 and 3 cases in TC and 15, 4 and 11 in TI were subgrouped, respectively (Fig. 4a), indicating that TI had a significantly higher expression of ALDH1A3 than TC (*P* < 0,001).

We observed ALDH1A3 expression in tumor cells (Fig. 2g, arrows), in outermost layer cells of some glomeruloid tufts (Fig. 2h, arrows), as well as in vessels (Fig. 2i, arrows). Some multiple-nucleus cells with dendritic-like structures (Fig. 2j, arrows) and small cells displaying glial-shaped morphology (Fig. 2j, arrowheads) also appeared ALDH1A3 positive. Of note, ALDH1A3 immunoreactivity was not detected in pseudopalisades (Fig. 2k, arrows) and necrosis centers (Fig. 2k, asterisk) in all investigated sections in this study. There was no apparent ALDH1A3 positive cells in control (Fig. 2l).

**Fig. 3:**
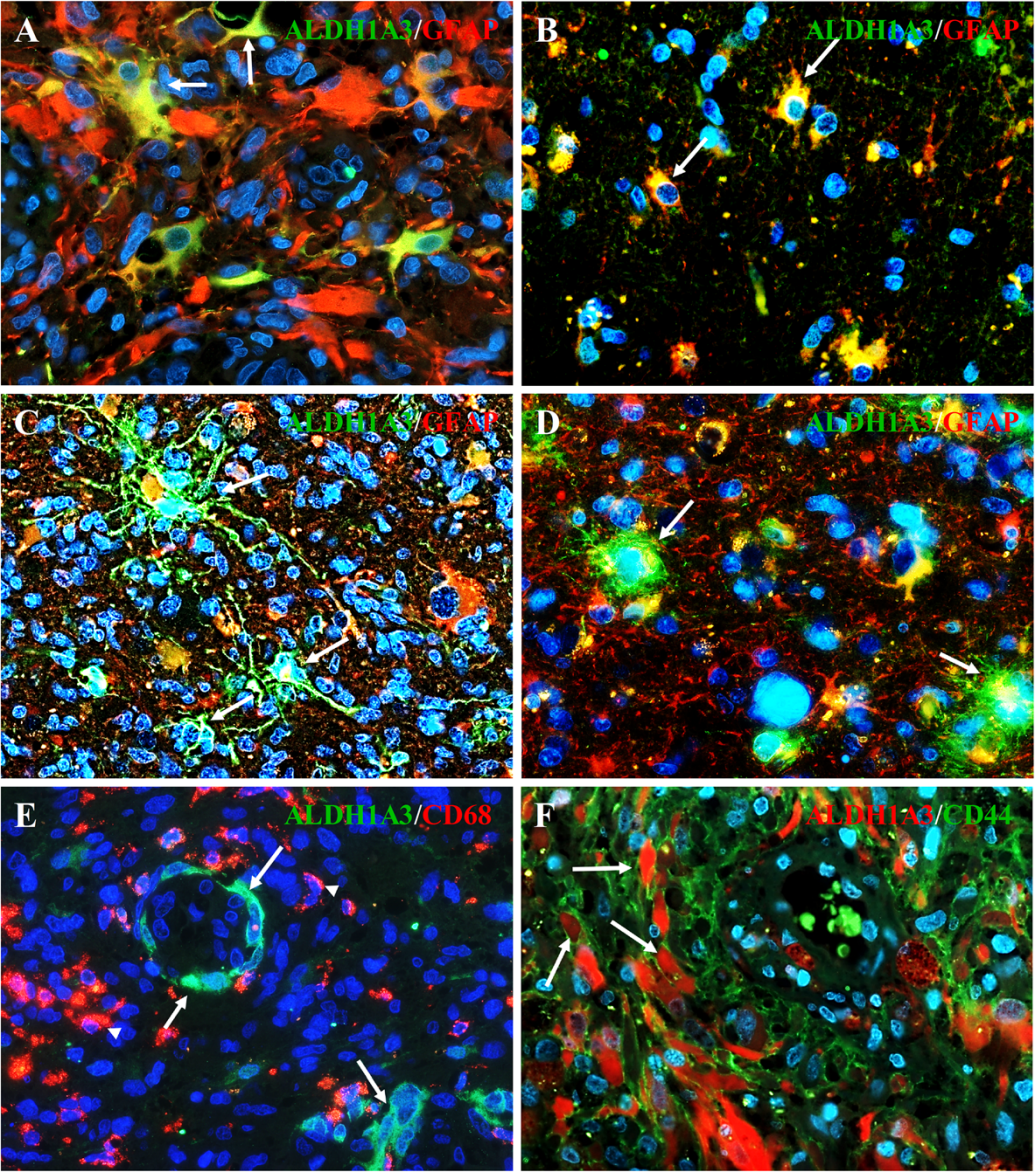
Double staining of ALDH1A3 with different markers in GBM sections. **a-d** Double staining of ALDH1A3 with GFAP. In both TC and TI, the co-expression of ALDH1A3 (green) and GFAP (red) was detected in some glial-shaped cells (arrows in **a**), tumor cells (arrows in **b**) and multi-nucleated cells (arrows in **c** and **d**). **e** Double staining of ALDH1A3 with CD68. ALDH1A3 immunoreactivity (green) detected in the outer layer of glomeruloid tuft (arrows in **e**) was negative for the macrophage marker CD68 (red, arrowheads). **f** Double staining revealed a coexpression (arrows) of ALDH1A3 (red) with the stem cell marker CD44 (green). **a-f**, original magnification × 400

In order to identify ALDH1A3 expressing cells, immunofluorescence staining of ALDH1A3 in combination with cell type specific markers was performed. Some glial-shaped cells and tumor cells were found double positive for ALDH1A3 and GFAP (Fig. 3a, b, arrows, respectively). Interestingly, the immunoreactivity of ALDH1A3 and GFAP was merged also in the dendritic-processed cells (Fig. 3c, arrows) and in cells with multiple nuclei (Fig. 3d, arrows). Cells expressing ALDH1A3 are often found positive for a stem cell surface marker CD44 (Fig. 3f, arrows), but not for a macrophage marker CD68 (Fig. 3e, arrowheads).

### Correlation of ALDH1A3 expression with clinical parameters

**Fig. 4:**
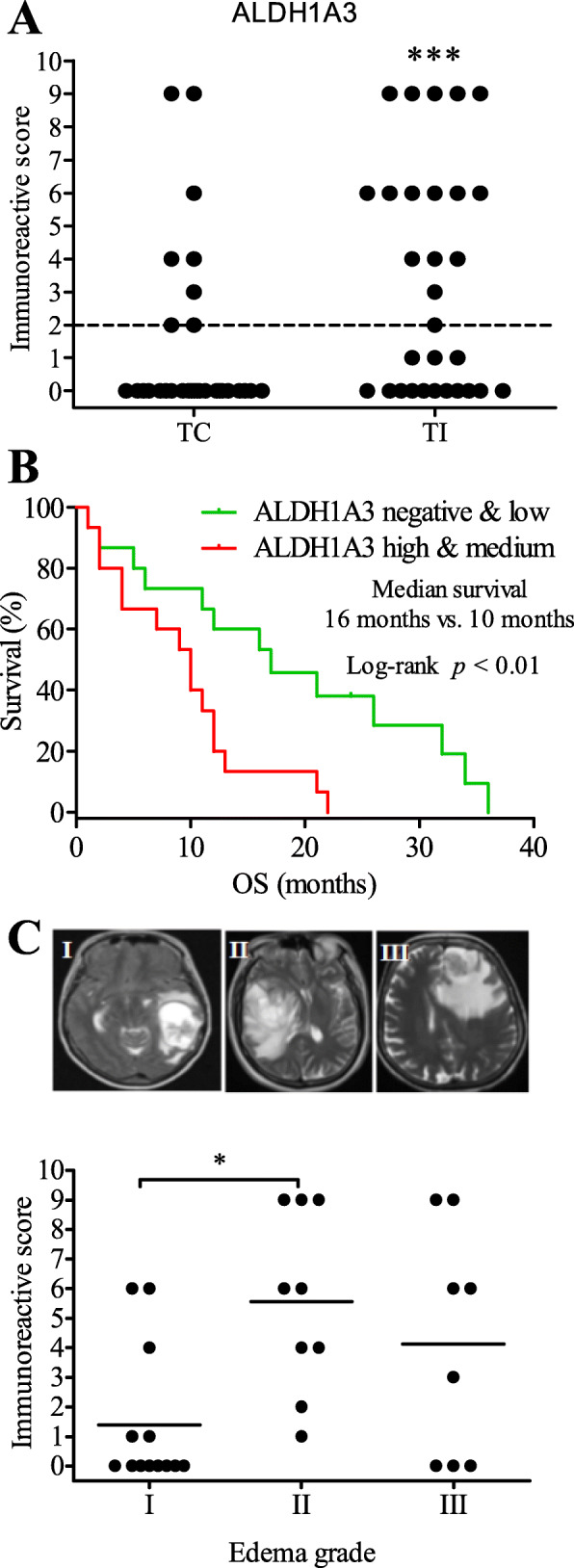
Association of ALDH1A3 expression with clinical parameters. **a** Quantitative analysis of ALDH1A3 immunoreactivity in TC and in TI of GBMs. The immunoreactive score (IRS) was evaluated in TC and TI as described in the method (*n* = 30). Based on IRS, TI had a significantly higher ALDH1A3 expression than TC (*P* < 0.001). In according to TI, GBM with IRS ≤ 2 was grouped as negative/low (*n* = 15), whereas GBM with IRS > 2 was defined as high and medium group (*n* = 15). **b** The association of ALDH1A3 expression with the OS period of patients. A higher expression of ALDH1A3 was significantly associated with a shorter OS time. **c** The representative MRI images show the different grades of edema. Peritumoral edema grade II had a higher IRS of ALDH1A3 compared with edema grade I. Paired t test was performed to compare IRS between TI and TC in **a** (***P < 0.001). One way ANOVA followed by Bonferroni’s multiple comparison test, comparing each column to all the other columns, was employed for multi-groups comparison in **c** (*P < 0.05)

According to IRS of ALDH1A3 in TI, the cohort of GBM patients was subgrouped as follows: negative/lower and medium/high expression of ALDH1A3 (Fig. 4a). The association of ALDH1A3 expression with clinical parameters in each subgroup was analyzed and summarized in Table 2. No statistical significance was found for the correlation of ALDH1A3 expression with all these analyzed parameters except OS and peritumoral edema. A higher expression of ALDH1A3 was significantly associated with a shorter median OS (*P < 0.01*, HR = 3.170, 95% CI: 1.328–7.566) (Fig. 4b). The median survival time of patients with medium/high ALDH1A3 expression was 10 months, while that of patients with negative/low ALDH1A3 group was 16 months. Furthermore, the higher expression of ALDH1A3 was also associated with a higher grade of peritumoral edema (*P* < 0.05, Fig. 4c). We further analyzed the association of MGMT promoter methylation status with OS, and no significant association was found in our patient cohort. The median survival in both MGMT promoter methylation positive (MGMT+, *n* = 14) and negative (MGMT−, *n* = 14) patients was 12 months. Next, we also studied association of OS with a combination of MGMT methylation status and ALDH1A3 expression. Of note, the median survival in the subgroup of MGMT+ with low ALDH1A3 IRS was found significantly longer than that in the subgroup of MGMT+ with higher ALDH1A3 IRS (17 months vs 7 months, respectively; *P* < 0.01). However, there was no significant difference in OS seen between low and higher ALDH1A3 expression in the subgroup of MGMT− patients (13.5 months vs 10.5 months).

**Table 2:** Analysis of the association of ALDH1A3 IRS with clinical parameters

Clinical parameter	Patient number	ALDH1A3		*P*
		negative/low (IRS ≤ 2)	medium/high (IRS > 2)	
Total patients	30	15	15	
Age				0,456^a^
< 60	12	7	5	
≥ 60	18	8	10	
Gender				0,464^a^
Male	14	6	8	
Female	16	9	7	
KPI				1,000^b^
≥ 70	26	13	13	
< 70	4	2	2	
SVZ				0,121^a^
+	20	12	8	
−	10	3	7	
Extent of Resection				0,456^a^
GTR	18	8	10	
Partial	12	7	5	
MGMT promotor methylation				0,464^a^
Yes	14	6	8	
No	14	9	7	
N/A	2	1	1	
IDH1 mutation				0,270^b^
Yes	3	2	1	
No	17	5	12	
N/A	10	8	2	
Standard chemoradiotherapy				0,256^a^
Yes	19	11	8	
No	11	4	7	
Ki67 index (%)	27	16,3 ± 3,1 (*n* = 12)	17,3 ± 2,0 (*n* = 15)	0,789^c^

*IRS* immunoreactive score, *KPI* Karnofsky Performance Index, *SVZ +/−* tumor contacted to subventricular zone (SVZ+) or contacted to non-subventricular zone (SVZ−), *GTR* gross total resection, *MGMT* O6-methylguanine-methyltransferase, *IDH1* isocitrate dehydrogenase 1, *N/A* not applicable

^a^Pearson chi-square test; ^b^Fisher’s exact test; ^c^Student t test with Welch’s correction

In addition, Ki67 index, a proliferation parameter, was 16.3 and 17.3% in low and higher ALDH1A3 subgroup, respectively (Table 2), and appeared as not associated with ALDH1A3 expression.

### Association of the expression of ALDH1A3 and signaling proteins in GBM

**Fig. 5:**
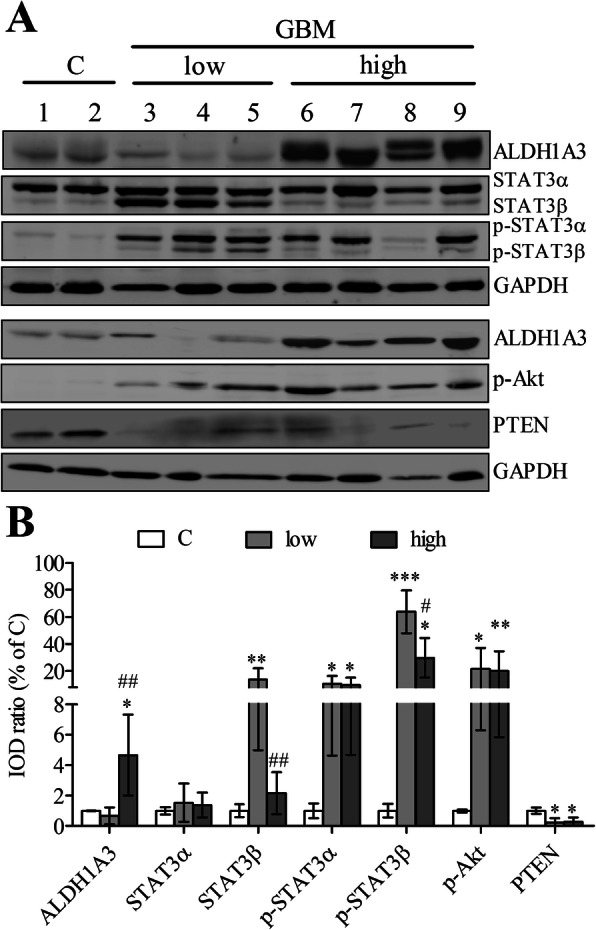
Detection of ALDH1A3 and the signaling proteins in GBM by western blot. **a** Representative blots detected the expression of STAT3 and p-STAT3, p-Akt, PTEN and GAPDH in the control (C, lane 1–2) and in GBM cases with lower immunoreactivity score (IRS ≤ 2, lane 3–5) and with higher immunoreactivity score (IRS > 2, lane 6–9) (See original images in Supplementary Fig. S1). **b** Semi-quantification of the blots. The integrated optical density (IOD) of the individual bands on blots was measured by ImageJ and the ratio of the target protein to the housekeeping protein GAPDH was calculated. The relative expression data were presented when the ratio of the control group was normalized as 1. One way ANOVA followed by Bonferroni’s multiple comparison test was employed for multi-groups comparison in **b**. *P < 0.05, **P < 0.01 and ***P < 0.001: compared with the control (C); #*P* < 0.05 and ##*P* < 0.01, compared with the group “low” (IRS ≤ 2)

Figure 5a shows representative blots detecting ALDH1A3, STAT3 and p-STAT3, p-Akt, PTEN and GAPDH in GBM and in control. Western blot confirmed distinct expression level of ALDH1A3 in subgroups of GBMs (*P* < 0.01). As expected, an upregulation of ALDH1A3 was confirmed in the GBM cases with higher IRS (IRS > 2, lane 6–9), whereas a low protein level of ALDH1A3 was detected in the GBM cases with lower IRS (IRS ≤ 2, lane 3–5). Semi-quantitative analysis of the blots revealed a significant upregulation of p-STAT3α, p-STAT3β and p-Akt accompanied by a downregulation of PTEN in GBMs compared to control. Interestingly, the expression of STAT3β (*P* < 0.01) and p-STAT3β (*P* < 0.05), but not STAT3α and p-STAT3α, was inversely associated with the protein level of ALDH1A3 in two subgroups of GBM (Fig. 5b).

## Discussion

Increasing evidence indicates ALDH1A3 as an important molecule that influences a diverse range of biological processes in CSCs and in tumor cells, thereby being associated with the initiation, progression, and prognosis of various cancers including GBM [27]. The present study investigated the expression pattern of ALDH1A3 at both mRNA and protein levels in human GBM specimens with emphasis on its association with clinical parameters of the patients.

Analysis of the microarray based TCGA-GBM dataset revealed that the mean level of *ALDH1A3* mRNA in the total cohort was lower than that in control. However, when considered in genomic subtypes of GBM [28, 29], its mRNA level appeared significantly higher in the MES than in the classical and proneural subtypes; even in the MES of GBM, the mean level of *ALDH1A3* mRNA was comparable with that in control (Supplementary Fig. S2). On the other hand, the TCGA-GBM dataset showed an inverse association of *ALDH1A3* mRNA expression with OS of GBM*.* These findings from the TCGA data encouraged us to further investigate implication of ALDH1A3 in GBM. In the present study, we demonstrated a significant downregulation of *ALDH1A3* mRNA expression in our GBM cohort in comparison to control, consistent with the findings derived from the TCGA dataset. As the cellular, biological functions of a molecule are ultimately determined by its translational/post-translational levels, not by its transcriptional level, we next focused on ALDH1A3 expression at protein levels in individual GBM tissues of our cohort. As shown in Fig. 2l, the immunoreactivity of ALDH1A3 was not detected in the control (normal) brain tissue. Furthermore, by western blot we also demonstrated a 4.66-fold higher protein expression of ALDH1A3 in the subgroup of GBMs with IRS > 2 than that in the control group (Fig. 5b). These findings allow us to postulate that a higher mRNA level of ALDH1A3 does not necessarily refer to its higher protein level. Indeed, several potential mechanisms regulating the translational and post-translation of ALDH1A3 have been identified: the hypermethylation status of ALDH1A3 promoter predicts a low expression of ALDH1A3 protein accompanied by a better prognosis of GBM patients [30]; USP9X-mediated deubiquitinase plays an important role in ALDH1A3 protein stabilization [31]; temozolomide (TMZ) treatment at high concentrations does not alter ALDH1A3 mRNA levels, but protein levels through autophagy [32]. Thus, it is more reliable to analyze the association of clinical parameters with ALDH1A3 protein levels.

GBM tumor is composed of heterogeneous cell populations including a small population, glioma CSCs [2], which is related to therapeutic resistance of GBM [3, 33]. As the subventricular zone (SVZ) is often proposed to be the source of CSCs [6, 8], we evaluated mRNA expression levels of *ALDH1A3* as well as other well-known CSC markers including *Nestin, CD133, CD44, YKL40, OLIG2, SOX2* in GBM. Despite a 2.5-fold higher *ALDH1A3* mRNA detected in the SVZ+ group, none of these investigated CSC markers showed a statistically significant alteration in mRNA levels (Fig. 1d). These results are consistent with previous reports [34, 35], the mechanism under which need to be further investigated.

The ALDH superfamily is the most important aldehyde metabolic enzyme family in human cells and has been linked to metabolism reprogramming in the initiation, metastasis, and recurrence of cancer [36]. Among the 19 members, the ALDH1A3 isoform has been served as the major source of total ALDH activity in GBM [15]. Thus, it is important to check not only *ALDH1A3* mRNA levels but also its protein levels that is more relevant to its enzymatic activity. Our immunohistochemistry study revealed a distinct expression of ALDH1A3 in individual GBM patients and a high inter-tumoral heterogeneity. A clear positive immunoreactivity of ALDH1A3 was detected only in 15 of 30 (50%) GBMs. Among these positive cases, 4 and 11 cases showed medium (IRS > 2) and high (IRS ≥ 6) expression of ALDH1A3, respectively. ALDH1A3 positive cells were mostly located at the tumor infiltrative area, suggesting that ALDH1A3 may participate in tumor cell invasiveness and metastasis. In fact, knockdown of ALDH1A3 expression in vitro models also suppressed cancer cell invasion in different entities [37, 38]. We demonstrated that a higher ALDH1A3 IRS was significantly associated with a short OS. As supporting, the expression of ALDH1A3 was positively associated with the grade of peri-tumoral edema that is also a prognostic parameter for GBM patients [39, 40]. Regardless of treatment, MGMT promoter methylation is an independent and favorable prognostic factor in GBM [41]. MGMT encodes a DNA-repair protein that inhibits the effect of treatment through removing alkyl groups from guanine, a target site for alkylating chemotherapy agents such as TMZ. MGMT promoter methylation is associated with higher therapeutic effect of TMZ and longer OS in GBM patients [42]. However, controversial results are also observed in several studies [43, 44]. In the present study, MGMT promoter methylation status was not associated with OS in GBM. Interestingly, when combining MGMT and ALDH1A3 expression together, low expression of ALDH1A3 may sensitively predict a better prognosis than those with higher expression of ALDH1A3 in the subgroup of MGMT+ patients. Hence, evaluation of ALDH1A3 expression might be a powerful prognostic tool in combination with MGMT promoter methylation status. IDH1 mutation is another prognostic maker for GBM. In TCGA database ALDH1A3 mRNA expression is negatively associated with IDH1 mutation [15]. In our cohort only 3 of 30 patients were identified with IDH1 mutation, and nevertheless, we did analysis of the association of ALDH1A3 expression with IDH1 mutation status. As predicted, no association was found between the level of ALDH1A3 protein or mRNA with IDH1 mutation. Therefore, a larger patient cohort is needed to verify the association of ALDH1A3 protein with IDH1 mutation in the future. Finally, we also observed the expression of ALDH1A3 frequently in the outer layer of different stage of glomeruloid tufts and in some endothelial cells of tumor vessels. In line with the pro-angiogenic functions of ALDH1A3 in vitro [45, 46], our findings by tissue staining also implicate ALDH1A3 in microvascular proliferation and in neo-angiogenesis in a subgroup of GBM. These clinical associations highlight ALDH1A3 as a potential prognostic biomarker and as a therapeutic target preventing tumor invasion and angiogenesis.

The TI consists of various cell types including infiltrating tumor cells, CSCs, reactive astrocyte, microglia, oligodendrocytes, inflammatory and immune cells, endothelial cells, and stromal cells thereby creating a complex microenvironment feasible for tumor growth and invasion as well as survival and therapy-resistance [47, 48]. To identify a cell type expressing ALDH1A3, we performed double staining of ALDH1A3 with the following cell type specific markers: GFAP, CD68 and CD44. Among ALDH1A3 positive cells, there were some GFAP positive cells likely reactive astrocytes or tumor cells due to their distinct morphology. As the co-expression of ALDH1A3 with the stem cell marker CD44 was also found, we propose that ALDH1A3 positive tumor cells might possess the stem cell-like properties involved in tumor progression and therapy resistance. Tumor-associated macrophages (TAMs) are highlighted in GBM due to the considerable size of their population (30–50%) [49]. But, the present study did not observe cells co-expressing ALDH1A3 and CD68. The possible implication of ALDH1A3 in immunoreaction mediated by immune cells needs to be further studied.

Along with important roles of ALDH1A3 in cancer and cancer stem cells, its underlying regulatory mechanism has become of interest. Several non-coding microRNAs including miR-600hg [50], miR-7 [51], miR-187 and miR-125a/b [27] have been identified to recognize ALDH1A3 as a target gene and to suppress ALDH1A3 expression. Forkhead Box D1 (FOXD1) regulates the transcriptional activity of ALDH1A3 in glioma stem cells (GSCs) of MES subtype [52]. The hedgehog pathway can significantly increase the expression of ALDH1 in cancer stem cells of ovary, which can also be a possible mechanism in GBM [53]. In a recent study, ALDH1A3 is stabilized upon ubiquitin-specific protease 9X (USP9X) in the MES GSCs. In contrast, deletion of USP9X induces degradation of ALDH1A3, and a subsequent decrease in p-STAT3, whereas overexpression of ALDH1A3 can restore p-STAT3 expression in GSCs in which downregulation of ALDH1A3 was induced by USP9X depletion [31]. On the other hand, in NSCLC CSCs, activation of STAT3 pathway significantly increases ALDH1A3 expression while multiple inhibitors of STAT3 signaling can decreased ALDH1A3 expression [17]. Taken together, we assume that there might be a feedback loop between ALDH1A3 and STAT3 to promote tumor progression in cancers. Developing STAT3 signaling inhibitors has been a new strategy for anticancer therapy. However, so far none of them is satisfied in the clinic studies [54]. STAT3 has two mainly isoforms, STAT3α and STAT3β, of which the latter is a suppressive factor of STAT3α due to lacking the transactivation domain [55]. In the present study, we found that ALDH1A3 protein expression was inversely associated with the levels of total STAT3β and p-STAT3β but no association was seen between the expression of ALDH1A3 and STAT3α. The association and the underlying regulatory mechanism of ALDH1A3 and STAT3 in GBM need to be further elucidated by using gene techniques in the future. Akt/PTEN is an important signaling implicated in numerous malignant tumors including GBM. We demonstrated a significant increase in p-Akt levels concomitantly accompanied by a downregulation of PTEN in GBM. However, the level of PTEN/p-Akt did not appear to be associated with ALDH1A3 expression in GBMs, suggesting that PTEN/Akt may not be a direct downstream signaling cascade trigged by ALDH1A3.

## Conclusion

The present study revealed a high intra- and inter-tumoral heterogeneity of ALDH1A3 expression manner in GBM. ALDH1A3 expression enriched in tumor infiltrative region highlights its crucial role in tumor invasiveness and progression. Moreover, we demonstrated an inverse association of ALDH1A3 expression with the prognosis of GBM, supporting ALDH1A3 as a prognostic marker and as a potential target for future GBM therapy.

## Supplementary information

**Additional file 1: Supplementary Figure S1.** Original blots of western blotting. **Supplementary Figure S2.** ALDH1A3 mRNA expression in subtypes of GBM and normal control based on TCGA database. Among three subtypes of GBM, ALDH1A3 mRNA level in the classical and proneural subtypes of GBM was significantly lower than that in the control and in the mesenchymal GBM. Student *t* test with Welch’s correction was used for data analysis between subgroups. **P < 0.01, ***P< 0.001, compared with control; ###*P* <0.001, compared with mesenchymal.

## Data Availability

All the data generated or analyzed during this study are included in this article.

## Abbreviations

ALDH1A3: Aldehyde dehydrogenase 1 family, member A3
ALDHs: Aldehyde dehydrogenases
CSCs: Cancer stem cells
FFPE: Formalin-fixed and paraffin-embedded
GAPDH: Glyceraldehyde 3-phosphate dehydrogenase
GBM: Glioblastoma
GFAP: Glial fibrillary acidic protein
IDH1: Isocitrate dehydrogenase 1
IRS: Immunoreactive score
KPI: Karnofsky performance index
MES: Mesenchymal subtype
MGMT: O6-methylguanine-methyltransferase
MRI: Magnetic resonance imaging
OS: Overall survival
PTEN: Phosphatase and tensin homolog
RT^2^-PCR: Real time reverse transcriptase polymerase chain reaction
STAT3: Signal transducer and activator of transcription 3
SVZ: Subventricular zone
TAMs: Tumor-associated macrophages
TC: Tumor center
TCGA: The cancer genome atlas
TD: Tumor distant area
TI: Tumor infiltrative area
TMZ: Temozolomide
