# Supplementary material for: The predominant expression of cancer stem cell marker ALDH1A3 in tumor infiltrative area is associated with shorter overall survival of human glioblastoma
Source: BMC Cancer. 2020 Jul 17;20:672. doi: 10.1186/s12885-020-07153-0 (PMC7368792; doi:10.1186/s12885-020-07153-0)
Supplement: Supplementary file 1 — Additional file 1: Supplementary Figure S1. Original blots of western blotting. Supplementary Figure S2. ALDH1A3 mRNA expression in subtypes of GBM and normal control based on TCGA database. Among three subtypes of GBM, ALDH1A3 mRNA level in the classical and proneural subtypes of GBM was significantly lower than that in the control and in the mesenchymal GBM. Student t test with Welch’s correction was used for data analysis between subgroups. **P < 0.01, ***P< 0.001, compared with control; ###P <0.001, compared with mesenchymal. [file 12885_2020_7153_MOESM1_ESM.docx]

**Supplemental Information**

**BMC cancer**

**The predominant expression of cancer stem cell marker ALDH1A3 in tumor infiltrative area is associated with shorter overall survival of human glioblastoma**

Chao Gan^1,3^, Daniela Pierscianek^1^, Nicolai El Hindy^1,4^, Yahya Ahmadipour^1^,

Kathy Keyvani^2^, Ulrich Sure^1^ and Yuan Zhu^1^

*^1^Department of Neurosurgery and Spine Surgery, University hospital Essen, University of Duisburg-Essen, Essen, Germany;*

*^2^Institute of Neuropathology, University hospital Essen, University of Duisburg-Essen, Essen, Germany;*

*^3^Department of Neurosurgery, Tongji Hospital, Tongji Medical College, Huazhong University of Science and Technology, Wuhan, China;*

*^4^Department of Spine- and Peripheral Nerve-Surgery, St. Christophorus Hospital, Werne, Germany*

Correspondence to Y. Zhu (yuan.zhu@uk-essen.de)


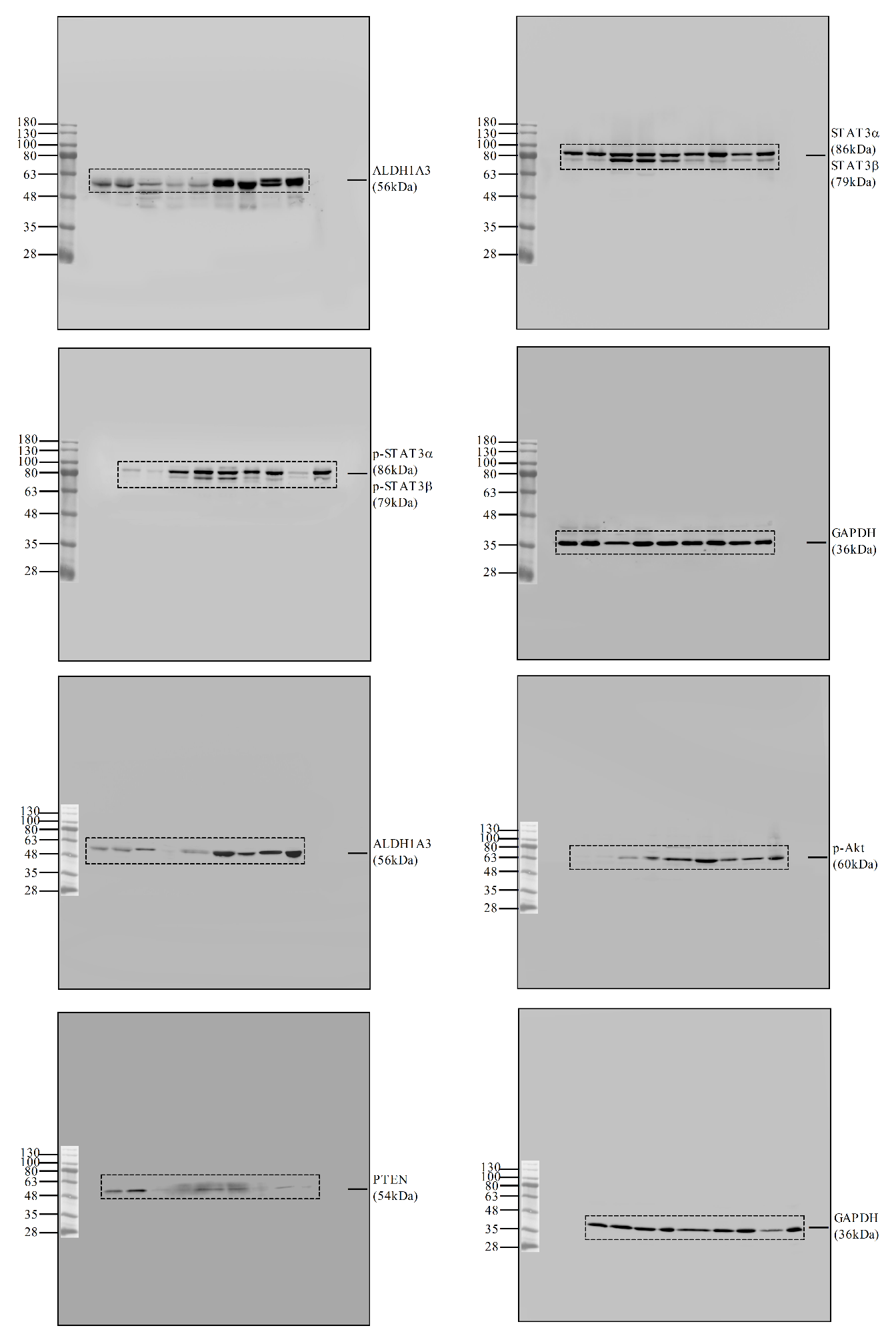
**Supplementary Fig. S1 Original blots of western blotting** (Corresponding to Fig. 5a in the manuscript)

**Supplementary**


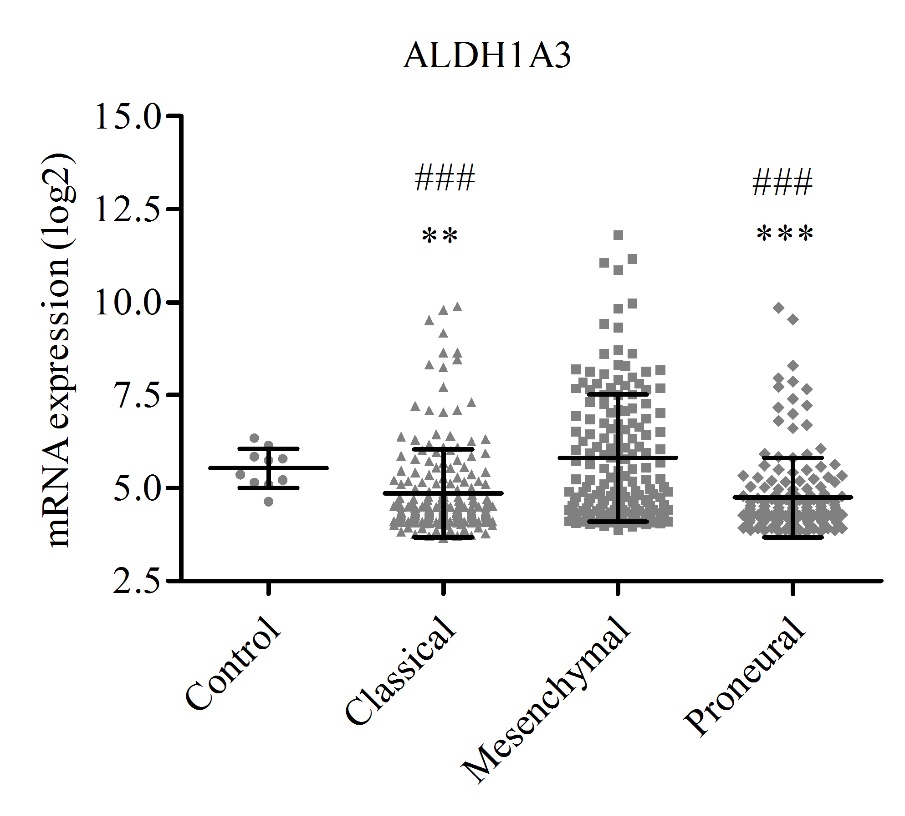


**Fig. S2 ALDH1A3 mRNA expression in subtypes of GBM and normal control based on TCGA database.** Among three subtypes of GBM, ALDH1A3 mRNA level in the classical and proneural subtypes of GBM was significantly lower than that in the control and in the mesenchymal GBM. Student *t* test with Welch’s correction was used for data analysis between subgroups. **P < 0.01, ***P< 0.001, compared with control; ###P <0.001, compared with mesenchymal.
